# Crown traits of coniferous trees and their relation to shade tolerance can differ with leaf type: a biophysical demonstration using computed tomography scanning data

**DOI:** 10.3389/fpls.2015.00172

**Published:** 2015-03-24

**Authors:** Pierre Dutilleul, Liwen Han, Fernando Valladares, Christian Messier

**Affiliations:** ^1^Environmetrics Laboratory, Department of Plant Science, McGill UniversityMontréal, QC, Canada; ^2^Museo Nacional de Ciencias Naturales, Consejo Superior de Investigaciones CientificasMadrid, Spain; ^3^Département des sciences biologiques, Centre d'étude de la forêt (CEF), Université du Québec à MontréalMontréal, QC, Canada; ^4^Département des ressources naturelles, Institut des Sciences de la Forêt tempérée (ISFORT), Université du Québec en OutaouaisRipon, QC, Canada

**Keywords:** plant light interception and shade tolerance, conifer crowns, needlelike vs. scalelike leaves, leaf area and volume, silhouette-to-total-area ratio (STAR), branching pattern complexity, fractal dimensions (FD), computed tomography (CT) scanning

## Abstract

Plant light interception and shade tolerance are intrinsically related in that they involve structural, morphological and physiological adaptations to manage light capture for photosynthetic utilization, in order to sustain survival, development and reproduction. At the scale of small-size trees, crown traits related to structural geometry of branching pattern and space occupancy through phyllotaxis can be accurately evaluated in 3D, using computed tomography (CT) scanning data. We demonstrate this by scrutinizing the crowns of 15 potted miniature conifers of different species or varieties, classified in two groups based on leaf type (10 needlelike, 5 scalelike); we also test whether mean values of crown traits measured from CT scanning data and correlations with a shade tolerance index (STI) differ between groups. Seven crown traits, including fractal dimensions (FD1: smaller scales, FD2: larger scales) and leaf areas, were evaluated for all 15 miniature conifers; an average silhouette-to-total-area ratio was also calculated for each of the 10 needlelike-leaf conifers. Between-group differences in mean values are significant (*P* < 0.05) for STI, FD1, FD2, and the average leaf area displayed (Ā_D_). Between-group differences in sign and strength of correlations are observed. For example, the correlation between STI and FD1 is negative and significant (*P* < 0.10) for the needlelike-leaf group, but is positive and significant (*P* < 0.05) for the miniature conifers with scalelike leaves, which had lower STI and higher FD1 on average in our study; the positive correlation between STI and Ā_D_ is significant (*P* < 0.05) for the scalelike-leaf group, and very moderate for the needlelike-leaf one. A contrasting physical attachment of the leaves to branches may explain part of the between-group differences. Our findings open new avenues for the understanding of fundamental plant growth processes; the information gained could be included in a multi-scale approach to tree crown modeling.

## Introduction

Individual plant light interception has been the subject of many studies in the last decades (see, e.g., Dutilleul et al., 2008; Duursma et al., 2012), because the amount of diffuse radiation intercepted for photosynthetic utilization by individual plants, and vegetation as a whole, plays an important role in growth and development, and hence productivity, as well as in atmospheric CO_2_ recycling and total carbon uptake (Ackerly and Bazzaz, 1995; Roderick et al., 2001; Sage and Coleman, 2001; Thornley, 2002). From both the agronomic and ecological perspectives, the relationship between light penetration (or its complement, light interception) and leaf area has been modeled with the Beer-Lambert law for light penetration into translucent media (Monsi and Saeki, 1953, 2005); see, e.g., Foroutan-pour et al. (2001), Duursma et al. (2012), and the references therein. A concept, or property of some plant species, related to light interception, is shade tolerance, originally proposed as the “capacity to endure shade” by Shirley (1943). While it is now generally acknowledged that shade tolerance indicates the degree to which a plant can survive and grow in low light conditions (Kobe et al., 1995), the survival, development, and reproduction of a plant species at a particular light level does not mean or imply that this species is at its physiological optimum (Humbert et al., 2007).

Crown traits important for plant light interception efficiency may be the same that influence shade tolerance. Among those traits, some can characterize the geometric structure and complexity of the branching pattern (fractal dimensions), and others, the amount of leaves (volume in 3D, number, areas in 2D). That is why, focusing on trees and coniferous species in particular, one of the objectives of our study was to investigate the existence of links and assess the strength and sign of correlations between various crown traits and a shade tolerance index, taking into account that there is no “classical” attachment of leaf blades to branches via petioles in conifers and leaf type for them can be of two types: needlelike or scalelike. In a detailed discussion of the architecture of terrestrial plants and their modular nature, the structural determinants of light capture and the 2-D and 3-D geometries of foliage arrangement within the crown, among other themes, Valladares and Niinemets (2007) emphasize that conifers have extensively aggregated foliage, citing Oker-Blom and Smolander (1988), Niinemets (1997), and Stenberg et al. (2001) for this.

Working at the whole-tree scale while collecting sufficiently fine data to measure crown traits accurately and thoroughly represents a challenge that we have addressed by applying high-resolution X-ray computed tomography (CT) scanning to the above-ground structure of miniature conifers, of less than 30 cm in width and height and growing in pots. Indeed, a CT scanner of medical type, like the one at the CT Scanning Laboratory for agricultural and environmental research on Macdonald Campus of McGill University, can be used for such small-size trees, and make indirect measurement of density on parallelepipedal rectangular parts (called voxels, the extension of pixels in 2D) of a size as small as 0.23 × 0.23 × 0.20 mm^3^ (width: 12 cm; height: 10 cm). Thus, the criterion of high resolution of Ketcham and Carlson (2001, Table 1) is satisfied, and more than 100 million data points (CT numbers) can be obtained for each individual tree. Recent applications of CT scanning technology in the plant sciences have been investigating the structural complexity of root systems (see, e.g., Gregory et al., 2003; Anderson and Hopmans, 2013), more than that of canopies or crowns (Dutilleul et al., 2005, 2008), and the fundamental question of tree growth using stem sections (see Dutilleul et al., 2014 and references therein).

Our objectives with the study reported here were multiple. Basically, we wanted to upgrade (analytically speaking) and expand (botanically speaking) the work of Dutilleul et al. (2008), who studied the developing crowns (branching patterns and leaf canopies) of four pyramidal (non-miniature) cedars (*Thuja occidentalis* Fastigiata). Leaving developing crown aspects aside but including plant light interception efficiency when possible, we aimed to (i) compare crown traits measured from CT scanning data between miniature conifers with needlelike vs. scalelike leaves, covering as wide a range of shade tolerance as possible; (ii) test for statistical differences between mean values of the crown traits and a shade tolerance index depending on leaf type; (iii) analyze correlations between crown traits and shade tolerance; and (iv) discuss possible biological meanings of (ii) and (iii). So doing, we tested the hypothesis of multi-fractality of branching pattern (Stewart, 1988; Prusinkiewicz and Lindenmayer, 1990), and provided supplementary data for the bottom right part of Figure 7 in Duursma et al. (2012), relating the average silhouette-to-total-area ratio (STAR) to the number of leaves; this number (*N*) is known to be large in conifers. All the abbreviations of variables that we have analyzed are defined, with their unit when they have one, in Table 1.

**Table 1:** **Abbreviated name of variables, their definition and unit**.

**Abbreviated name**	**Definition**	**Unit**
STI	Shade tolerance index	–
LA	Leaf area in vertical projection of the crown	mm^2^
PCT	LA divided by the area of the smallest disc including the vertical projection of the crown	%
FD1	Fractal dimension estimated over smaller scales	–
FD2	Fractal dimension estimated over larger scales	–
*V*_L_	Total leaf volume	mm^3^
*V*_L_ / *V*_B_	Leaf volume-to-branch volume ratio	mm^3^ mm^−3^
*N*	Estimated number of leaves	–
Ā_D_	Average leaf area displayed	mm^2^
*A*_L_	Total leaf area	mm^2^
STAR	Average silhouette-to-total-area ratio	mm^2^ mm^−2^
BA	Basal area	mm^2^

## Materials and methods

### Plant material

**Figure 1:**
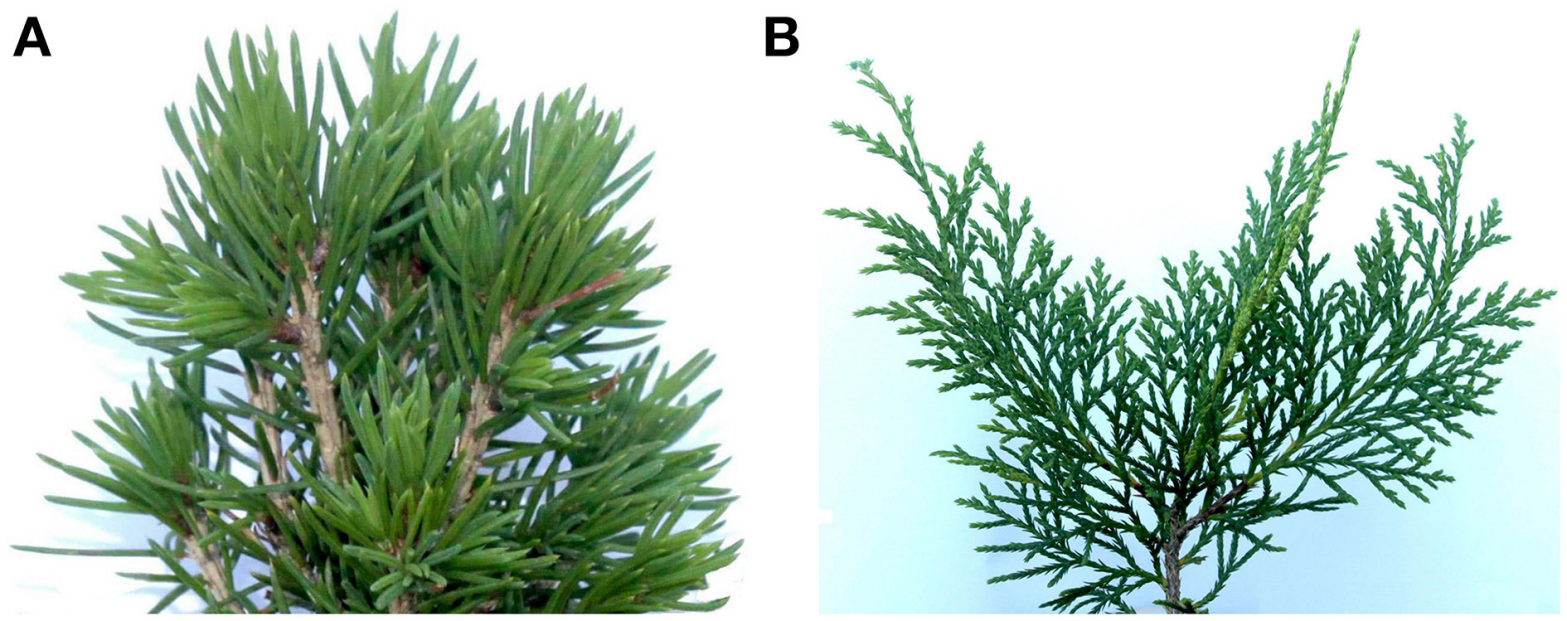
**Photographs of a portion of the crown of two of the 15 miniature coniferous trees**. **(A)** *Picea abies* Tompa (Norway Spruce) and **(B)** *Microbiota decussata* Gold Spot (Russian Arborvitae), as representative examples of the needlike and scalelike leaf groups, respectively.

The scientific and common species names of the 15 miniature conifers that we studied are listed in Table 2, where they are classified according to their leaf type (i.e., 10 needlelike, 5 scalelike; see Figure 1 for examples). These conifers, which are horticultural varieties of indigenous species, were grown in the Canadian Province of British Columbia (Pacific Northwest Propagators Inc., Rosedale) and purchased through a Montréal (Québec) plant shop in the month of May, prior to the scanning of their crowns in June-July (see below). Their height and width then varied, respectively, from 7.9 to 22.2 cm and from 10.3 to 27.5 cm, respectively. Trees were about 2 years old at the time of purchase. The rooting medium was organic, fertilized with Polyon NPK 16-3-13 plus minors.

**Table 2:** **Scientific and common species names of the 15 miniature conifers, their leaf type and shade tolerance index value, and the estimates and associated standard errors of the two fractal dimensions of their branching pattern**.

**Scientific name**	**Common name**	**Leaf type**	**STI**	**FD1 (std. error)**	**FD2 (std. error)**
*Abies balsamea* Nana	Dwarf Balsam Fir	Needlelike	4	1.0639 (0.0200)	1.6247 (0.1372)
*Cryptomeria japonica* Compressa	Japanese Cedar	Needlelike	3	1.1996 (0.0759)	2.0610 (0.0574)
*Cryptomeria japonica* Gyokuryu	Japanese Cedar	Needlelike	4	1.1585 (0.0381)	1.6351 (0.0517)
*Cryptomeria japonica* Monstrosa Nana	Japanese Cedar	Needlelike	4	1.3027 (0.0827)	1.9202 (0.0408)
*Juniperus horizontalis* Blue Pygmy	Creeping Juniper	Needlelike	2.5	1.2516 (0.0819)	2.0387 (0.1023)
*Picea abies* Thumbelina	Norway Spruce	Needlelike	4.5	1.0847 (0.0235)	1.5994 (0.0718)
*Picea abies* Tompa (^*^)	Norway Spruce	Needlelike	4.5	1.0934 (0.0400)	1.4709 (0.0731)
*Picea glauca* Cy's Wonder	White Spruce	Needlelike	3	1.1568 (0.0583)	1.9522 (0.0496)
*Picea glauca* Pixie	White Spruce	Needlelike	3	1.3465 (0.1120)	2.0049 (0.0121)
*Picea sitchensis* Papoose	Dwarf Sitka Spruce	Needlelike	3	1.1171 (0.0442)	1.6678 (0.1227)
*Chamaecyparis lawsoniana* Ellwood's Nymph	Port Orford Cedar	Scalelike	2.5	1.4507 (0.1368)	2.2796 (0.0223)
*Chamaecyparis obtusa* Tempelhof	Hinoki Falsecypress	Scalelike	3.5	1.5781 (0.1322)	2.0323 (0.0235)
*Chamaecyparis pisifera* Golden Pin Cushion	Sawara Falsecypress	Scalelike	1.5	1.4252 (0.1223)	2.1993 (0.0365)
*Juniperus chinensis* Shimpaku	Chinese Juniper	Scalelike	1.5	1.2741 (0.0186)	1.8288 (0.0571)
*Microbiota decussata* Gold Spot (^*^)	Russian Arborvitae	Scalelike	2.5	1.5176 (0.1319)	2.0507 (0.0396)

* Depicted in Figure 1.

### Shade tolerance index

As mentioned in the Introduction, plant tolerance to low light conditions, for survival and development, must be distinguished from optimum sun exposure, as recommended in horticulture for example. Accordingly, the shade tolerance index values for the varieties studied in Table 2 are equal or close to the values reported for indigenous species in forest ecology data sources (e.g., *Sylvics of North America*, Burns and Honkala, 1990; see also Kwantlen Polytechnic University, 2012). Values of our index range from 1.0 (requires Full Sun) to 5.0 (well-adapted to Full Shade), by increments of 0.5; the median value of 3.0 thus corresponds to: needs Half Sun/accommodates Half Shade.

### Computed tomography scanning

The 15 crowns of miniature conifers were CT scanned at the CT Scanning Laboratory for agricultural and environmental research on Macdonald Campus of McGill University in Ste-Anne-de-Bellevue (Québec, Canada), which is equipped with a helical high-resolution CT scanner XVision (Toshiba Corporation, Medical Systems Division, Tokyo, Japan). CT scanning sessions were distributed over several days, with two or three trees CT scanned per day. Basic CT scanning configuration parameters were the same for all 15 trees: tube current, 50 mA, and tube voltage, 120 kV. The X-ray beam width (i.e., a parameter to be distinguished from the thickness of CT images) was the same (1 mm) for all but one (the widest tree, for which 2 mm was used).

Depending on the width of the crown, a different field of view was used: SS, very small (18 cm in diameter); S, small (24); or M, medium (32). A zoom factor was used to improve spatial resolution horizontally; for example, for the crown of *Chamaecyparis pisifera* Golden Pin Cushion (Sawara Falsecypress), which had a width of 10.3 cm, a zoom factor of 1.5 was used with the SS field of view. Among the 11 miniature conifers that were CT scanned with the SS field of view, a zoom factor (ZF) was used for six (two times with *ZF* = 1.5 and four times with *ZF* = 1.2). Vertically, the thickness of CT images was 0.2, 0.3, or 0.4 mm, depending on the height of the tree; its width was also taken into account in order to have the same resolution as much as possible in all three dimensions (e.g., 0.23 × 0.23 × 0.20 mm^3^ for Sawara Falsecypress). Between 400 and 600 CT images, each made of 512 × 512 CT numbers, were produced. Of all the CT images produced, a small portion (the top ones) corresponded to pure air and another small portion (the bottom ones) contained information about the base of the tree and surface soil and roots.

Prior to CT scanning, the equipment was calibrated with the appropriate phantoms, so that the CT numbers for air and water corresponded to −1000 and 0 Hounsfield units (HU), respectively. Following CT scanning, the raw data files, which contained between ca. 100 and 150 million CT numbers in 512 × 512 matrices, were transferred to a Windows 7 Dell workstation for graphical and numerical analyses in MATLAB R2014a (The MathWorks Inc.).

### Fractal dimension estimation

For reasons already made clear when complexity of the above-ground structure was analyzed from photographs of plants from which leaves had been removed manually (i.e., the thickness of branches introduces a bias; Foroutan-pour et al., 1999a), fractal dimension estimation in our study was performed on skeletal branching patterns, prepared in a customized MATLAB graphical unit interface by tracing branches using the 3-D array of CT scanning data collected for the crown of a miniature conifer. The fact that branching patterns were skeletal means that their thickness was one voxel (i.e., the 3-D extension of one pixel in 2D); in our case, a voxel has two opposite square faces, and is the smallest volumetric unit for which a CT number is produced.

**Figure 2:**
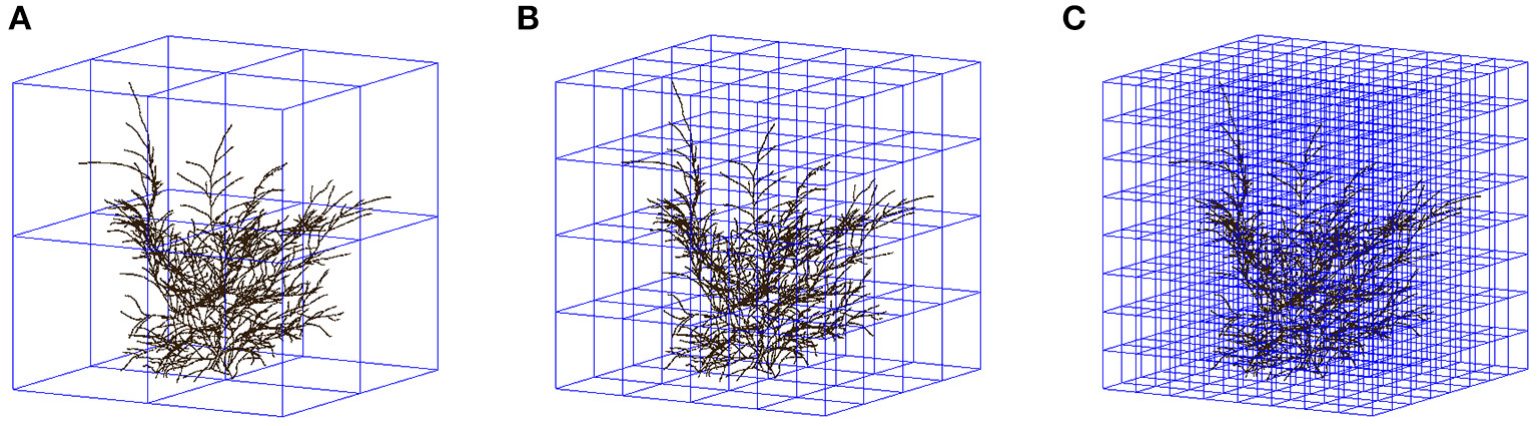
**Illustration of three successive steps in the cube-counting procedure of fractal dimension estimation, i.e., the steps in which one of the smallest cubes to contain the entire skeletal branching pattern of a miniature coniferous tree (here, *Microbiota decussata* Gold Spot) is divided into (A) 8 = 2 × 2 × 2; (B) 64 = 4 × 4 × 4; and (C) 512 = 8 × 8 × 8 cubes which have a sidelength equal to (A) 1/2; (B) 1/4; and (C) 1/8 of the sidelength of the start cube (438)**. The counting of the cubes that have a non-empty intersection with at least one branch segment provides *C*(*s*) at the corresponding sidelengths and scales in Equation (1); see also the 2nd, 3rd, and 4th data points from left to right in Figure 6B, for log(1/*s*) = log(1/219), log(1/110), log(1/55) (after rounding of *s* to the nearest integer) and log(*C*(*s*)) = log(8), log(38), log(150), respectively.

A cube-counting procedure was used to estimate fractal dimensions; for reasons that will appear clearly in the Results, two fractal dimensions (denoted FD1, FD2) were estimated for each tree. In the cube-counting procedure for fractal dimension estimation of an object or a structure in 3-D space, the object or structure of interest (e.g., a skeletal branching pattern) first needs to be included in the smallest cube that can contain it. In our framework, the length of the sides of that cube is given by the larger of two quantities: the number of horizontal sections containing the 3-D image of the skeletal branching pattern and the diameter (in voxels) of the smallest circle containing its vertical projection (along the Z-axis, onto the X-Y plane). For the two examples which will be detailed, that length is a perfect 400 (which can easily be divided by powers of 2, without rounding) and 438 (resulting in 219, 109.5, 54.75, 27.375, 13.6875, 6.84375, 3.421875, 1.7109375 after successive divisions by 2, rounded to 219, 110, 55, 27, 14, 7, 3, 2). These nine decreasing cube sidelengths, denoted “*s*” hereafter, provide as many scales, larger or smaller. The number of cubes with sidelength *s* intersecting a skeletal branching pattern was thus counted for 9 scales; Figure 2 illustrates the cube-counting procedure for the three cube sidelengths or scales corresponding to the divisions by 2, 4, and 8. In our customized MATLAB program, cube counting for a given scale was repeated 8 more times, by moving the “smallest cube containing the entire structure of interest” one voxel on the left/right, or one voxel in the front/back, or the two together. The minimum count for cube sidelength *s* (denoted “*C*(*s*)” below) was retained for a more accurate estimation, in accordance with similar procedures in 2D (Foroutan-pour et al., 1999b) and 3D (Lontoc-Roy et al., 2006); see also Li et al. (2009). A fractal dimension estimate is then the estimated slope of the straight line fitted by least squares in the biplot of log(*C*(*s*)) against log(1/*s*) over a number of scales, where log(.) is the natural logarithm and *C*(*s*) denotes the number of cubes with sidelength *s* intersecting the skeletal branching pattern:

(1) log(C(s))=k+FD log(1/s)

As we shall see in Section Bifractality of Conifer Branching Patterns, the *R*^2^-values in these fittings, depending on the range of scales covered, are very important.

### Plant light interception efficiency

**Figure 3:**
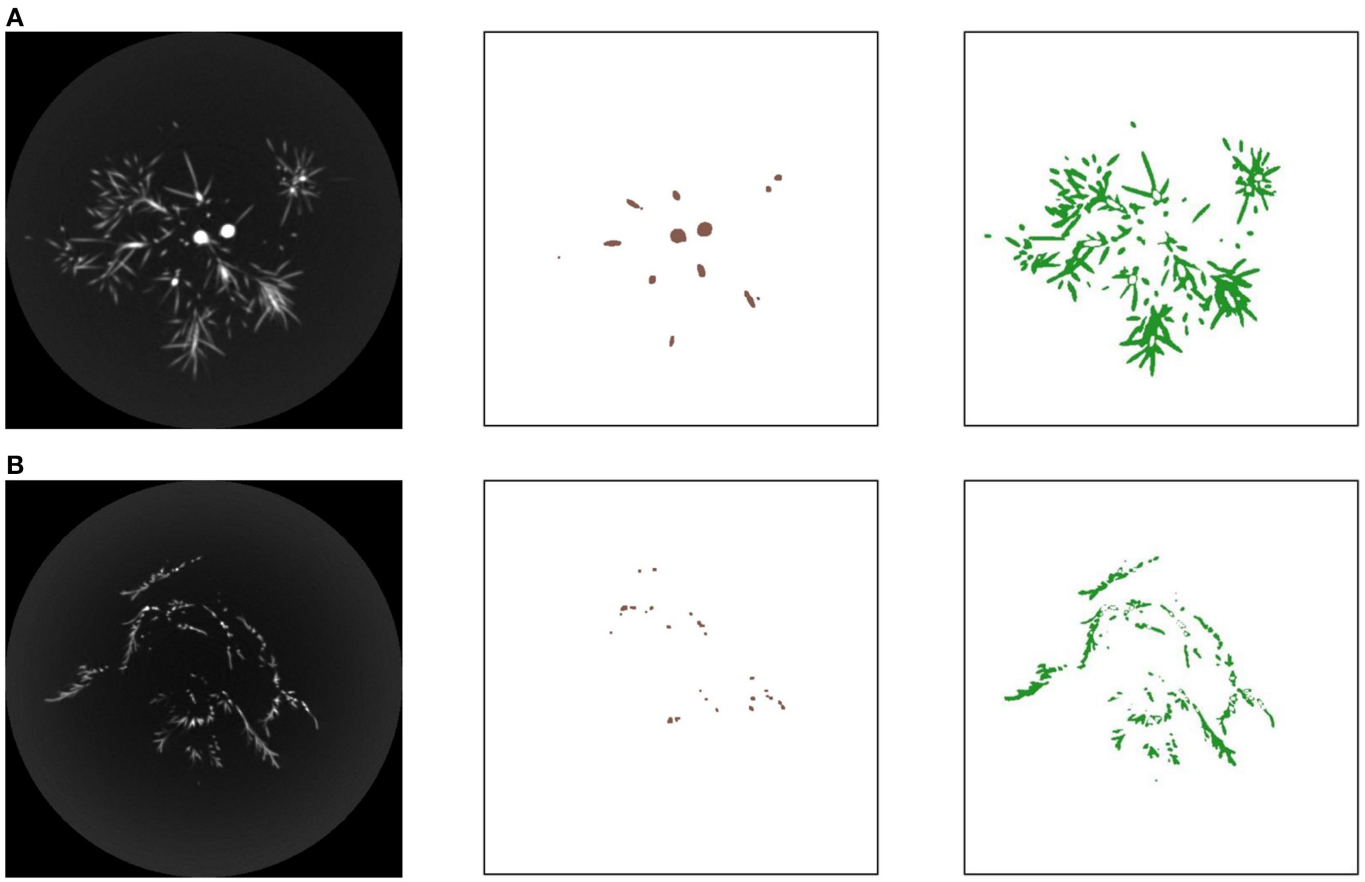
**(A)** From left to right, one of the 428 gray-tone CT images (thickness: 0.4 mm) constructed for the crown of the miniature coniferous tree (*Picea abies* Tompa) in Figure 1A; the branch part delineated from the corresponding 512 × 512 matrix of CT numbers, colored in brown; and the leaf part obtained by subtraction (excluding air) and colored in green. **(B)** Similar information for the miniature coniferous tree (*Microbiota decussata* Gold Spot) depicted in part in Figure 1B; the gray-tone CT image displayed is one out of 450 with thickness of 0.3 mm used to analyze the crown in this case. Note: The two skeletal branching patterns are contained in 400 and 438 CT images, respectively.

Besides the shade tolerance index (STI, obtained independently) and the two fractal dimensions (FD1, over smaller scales; FD2, over larger scales), five crown traits were evaluated from the raw CT scanning data or the derived 2-D or 3-D images, for all the 15 miniature conifers. These five crown traits are: absolute leaf area (LA, in mm^2^) in the vertical projection of the crown; relative leaf area (PCT, in %) in the smallest disc including the vertical projection of the crown; total leaf volume (*V*_L_, in mm^3^), obtained from all the leaf voxels (i.e., voxels with a CT number smaller than the branch threshold, and greater than −980 HU to discriminate them from air; see Figure 3 for examples); leaf volume-to-branch volume ratio (*V*_L_/*V*_B_); and average leaf area displayed (Ā_D_, in mm^2^; Pearcy et al., 2011; Duursma et al., 2012), over four classes of azimuth (the X- and Y-axes in the CT scanning framework and their two directions) × 20 solar elevation classes (every 4.5 degrees starting from 0), plus the 90-degree solar elevation.

For each of the 10 miniature conifers with needlelike leaves, we applied a cylindrical model (height, *h*; radius, *r*) to 25 leaves sampled in the crown on computer, using a random stratified design (1 stratum = 20% of the height of the tree, from bottom to top; 5 leaves randomly sampled and measured per stratum) and the 3-D array of CT numbers and the 3-D image of leaf voxels. This application consisted in equaling the measured individual leaf volume from CT scanning data with the volume of a cylinder, π *r*^2^ *h*, using the largest distance calculated in MATLAB between two voxels of the leaf for *h*, and solving the equality for *r*, which then allowed the calculation of an individual leaf area, π r^2^ + 2 π *r h*, excluding the bottom area of the cylinder (where the needlelike leaf is attached to the branch). Dividing the total leaf volume by the mean of the 25 individual leaf volumes thus provided an estimate of the total tree leaf number, *N*, which multiplied by the mean of the 25 individual leaf areas provided in turn an estimate of the total tree leaf area, *A*_L_ (in mm^2^). Finally, an average silhouette-to-total area ratio, STAR (mm^2^ mm^−2^) was calculated by dividing Ā_D_ by *A*_L_ (Oker-Blom and Smolander, 1988; Duursma et al., 2012).

**Figure 4:**
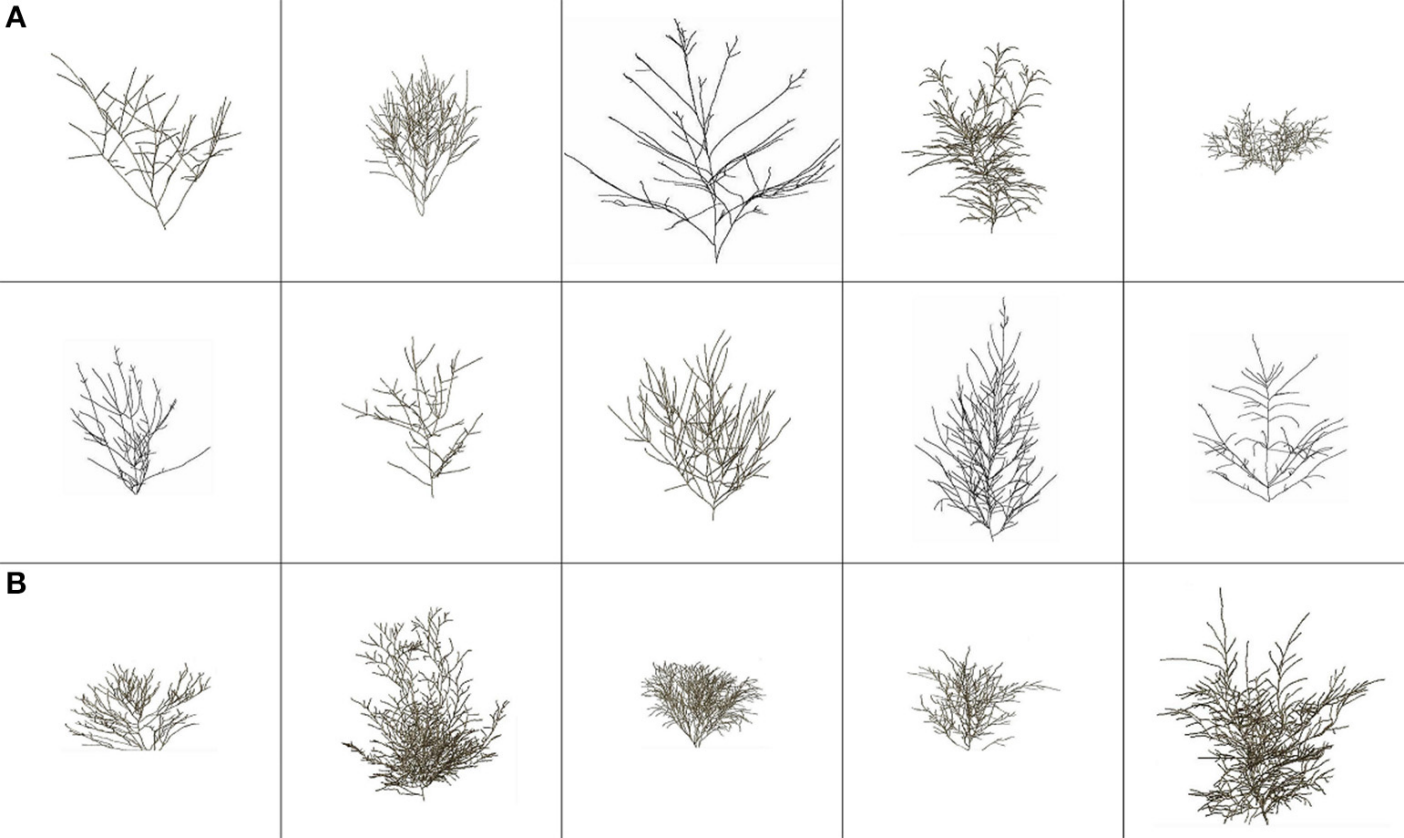
**Skeletal branching patterns (i.e., branches are represented with a one-voxel thickness) for the 15 miniature conifers (A) with needlelike leaves (first two rows) and (B) with scalelike leaves (third row), following the order of Table 2 from left to right instead of top-bottom; for example, the top left panel shows *Abies balsamea* Nana (Dwarf Balsam Fir), while the top right one shows *Juniperus horizontalis* Blue Pygmy (Creeping Juniper)**. Width and height (cm) values (for complete crowns; see Figure 5) range from 10.3, 7.9 (*Chamaecyparis pisifera* Golden Pin Cushion, Sawara Falsecypress) to 27.5, 22.2 (*Cryptomeria japonica* Gyokuryu, Japanese Cedar), respectively. These 3-D renderings and those of Figure 5 were constructed from CT scanning data, and conserve the relative differences in size (width and height) among the 15 coniferous trees.

No standardization was applied to *N* in Figure 7A of Duursma et al. (2012), for the investigation of a negative relationship with STAR. Nevertheless, it is a natural question to ask whether a standardization, e.g., by expressing size trait data (LA, *V*_L_, *N*, Ā_D_, *A*_L_) per unit stem basal area (BA), would change correlations. For our miniature conifers, we thus measured BA at a height of about 2.5 cm, starting from the first CT image in the 3-D skeleton of the branching pattern, as an equivalent to breast height in non-miniature trees; as specified in the legend of Figure 4, the height of our 15 miniature conifers ranges from 7.9 to 22.2 cm. The number of stem voxels found in the CT image at ca. 2.5-cm height, multiplied by the horizontal area of a voxel in mm^2^, provided the BA measure. The calculation of BA was made in the same way whether the miniature conifer had one or several stems; three of the 15 miniature conifers had several stems.

### Statistical analyses

Normality of the distribution of sample data was tested per leaf-type group for a given variable. It was accepted at 5% after arcsine-square-root transformation for PCT, after log-transformation for BA and without transformation for the other variables for which the mean values were compared statistically between leaf-type groups. Accordingly, these comparisons were carried with parametric *t*-tests, using effective degrees of freedom when the sample variances could not be pooled. Spearman's rank-based coefficient was used for correlation analyses because it can capture non-linear relationships, i.e., it is not restricted to linear relationships like Pearson's sample correlation coefficient. A *t*-test for paired observations was performed to compare the mean values of FD1 and FD2 over all the 15 miniature conifers and per leaf-type group. The SAS 9.3 (SAS Institute Inc.) procedures UNIVARIATE (option NORMAL), TTEST and CORR (option SPEARMAN) were used for the normality tests, *t*-tests for comparisons of means and correlation analyses, respectively.

## Results

### Conifer crown image processing

**Figure 5:**
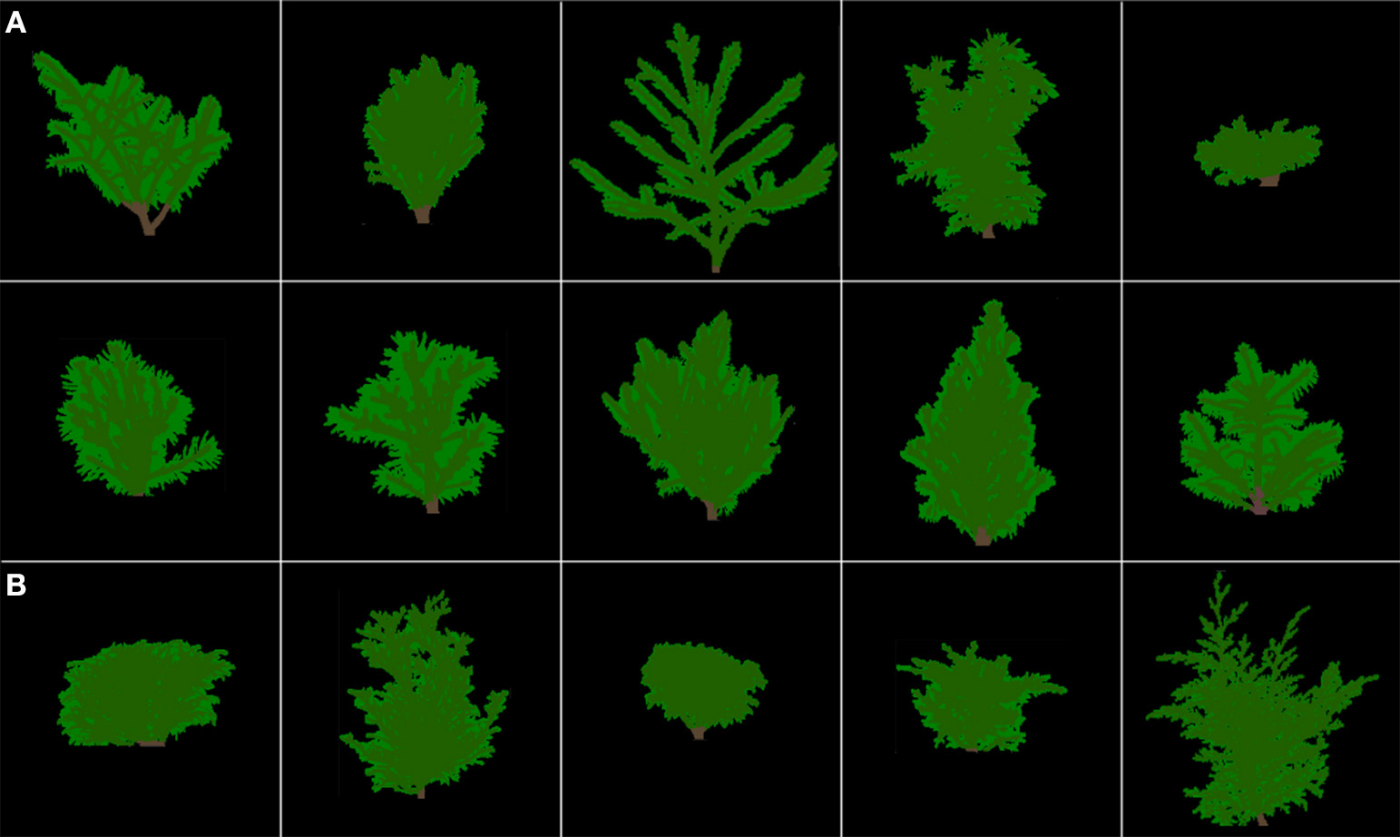
**False-colored 3-D renderings of the complete crowns (branches in brown, leaves in green) for the 15 miniature conifers of Figure 4, in same order and size representation**. **(A)** with needlelike leaves (first two rows) and **(B)** with scalelike leaves (third row).

The 15 skeletal branching patterns, which were traced from CT scanning data first, are displayed in 2D (front view) in Figure 4. Their actual 3-D structure can be better viewed with a customized MATLAB graphical unit interface, but differences in structural complexity among some of the crowns can already be anticipated prior to any quantification with fractal analysis (see Subsection Bifractality of Conifer Branching Patterns). After the appropriate number of layers was added to the branch skeletons, using −650 HU as threshold for most trees to delineate (from the CT numbers) branches from leaves attached to them, entire “digital branches” were obtained (see middle images in the detailed examples of Figure 3) and thereafter, by subtraction (excluding air) remained leaves (see right images in the detailed examples of Figure 3). This way of proceeding at the whole-tree scale provided the complete crown renderings of Figure 5, with a semi-transparency option to allow the eye to penetrate the leaf canopies. Again, prior to any quantification through leaf areas and volumes (see Subsections Differences in Means of Shade Tolerance Index and Conifer Crown Traits and Light Interception Efficiency for Needlelike-Leaf Group), differences among some of the crowns can be anticipated in light interception efficiency.

### Bifractality of conifer branching patterns

**Figure 6:**
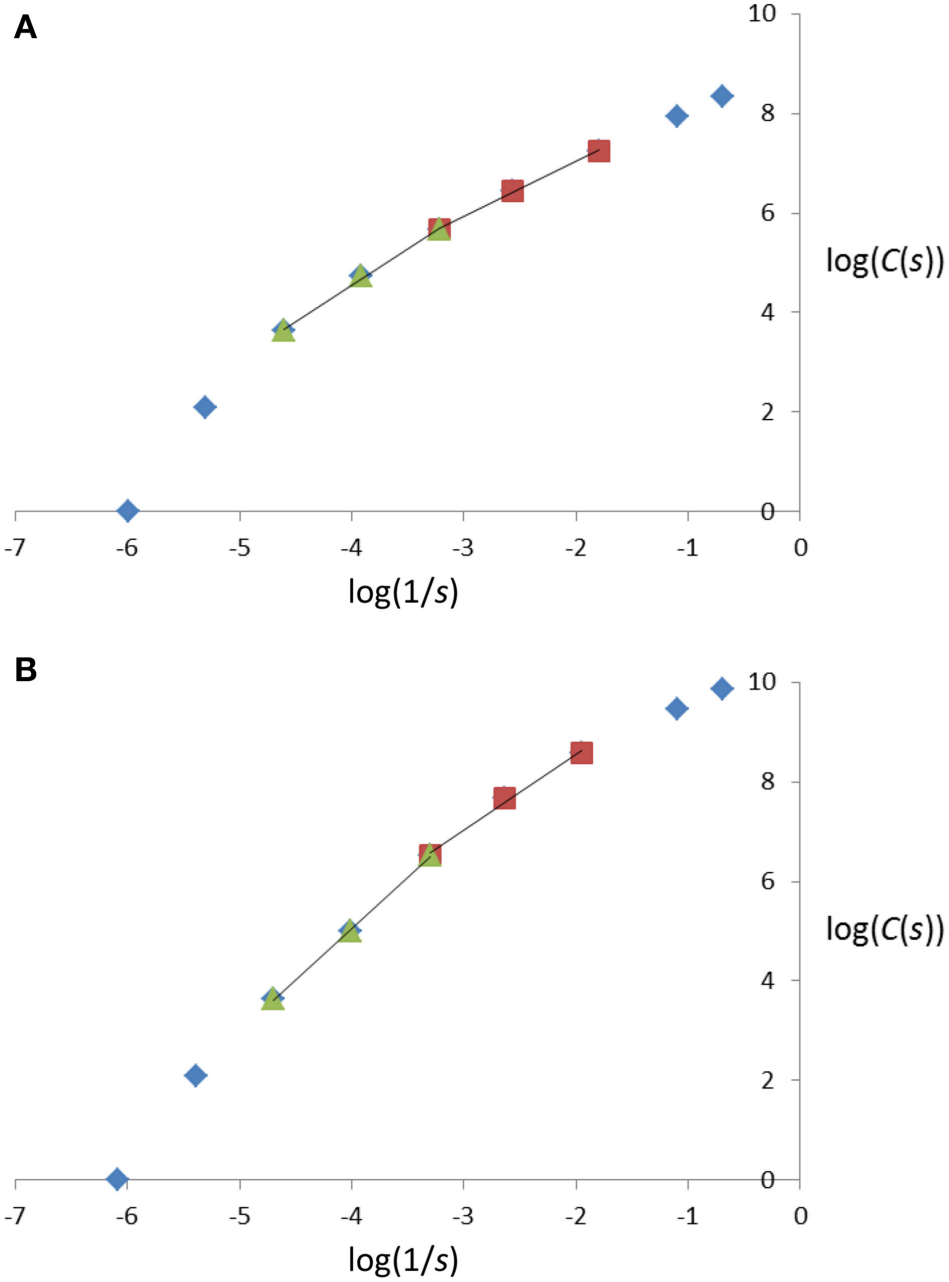
**Plot of log(*C*(*s*)) against log(1/*s*), with *s* representing the cube sidelengths used in the cube-counting procedure of fractal dimension estimation, and *C*(*s*), the number of cubes with sidelength *s*, for the two miniature coniferous trees, (A) *Picea abies* Tompa and (B) *Microbiota decussata* Gold Spot, in Figures 1A, 3A and 1B, 3B, respectively**. See text for details about the estimation procedure.

As a preliminary note, it is important to emphasize that in log-log plots such as those of Figure 6, where log(*C*(*s*)) is plotted against log(1/*s*), the smaller scales *s* (i.e., smaller cubeside lengths, after divisions by the greater powers of 2 in the cube-counting procedure) are represented by data points on the right, and the larger scales *s* (i.e., larger cubeside lengths, after divisions by a few powers of 2 in the procedure), by data points on the left. This positioning (smaller scales right, larger scales left) follows from the use of the inverse in log(1/*s*) and the fact that logarithmic functions take negative values for positive quantities smaller than 1.0. Accordingly, we number the scales from right to left (i.e., from smaller scales to larger scales) below.

Following Foroutan-pour et al. (1999b), it is not recommended to include the smallest scales (1 and 2 here; see, e.g., the top-right data points in Figure 6) and the largest scales (8 and 9; see, e.g., the bottom-left data points in Figure 6) in a box-counting procedure of fractal dimension estimation, because the estimation would be biased if they were included. Remain then the options of using all five middle data points (scales 3-4-5-6-7) and subsets of four and three successive data points (scales 3-4-5-6, 4-5-6-7 and 3-4-5, 4-5-6, 5-6-7). We tried them all and found that the FD estimates obtained using Equation (1) with five scales and four scales had intermediate values, between those for scales 3-4-5 (smaller scales) and 5-6-7 (larger scales), and were close to the FD estimates for scales 4-5-6. More concretely, for the examples of Figure 6, the FD estimates (with the associated *R*^2^-value as measure of goodness-of-fit in parentheses) read as follows (in the same order of subsets of scales as above): 1.2749 (0.9920), 1.1744 (0.9962), 1.3729 (0.9952), 1.0934 (0.9987), 1.2611 (0.9984), 1.4709 (0.9975) in Figure 6A and 1.8218 (0.9923), 1.7299 (0.9883), 1.9739 (0.9988), 1.5176 (0.9925), 1.946 (0.9971), 2.0507 (0.9996) in Figure 6B.

Because (i) there is a change in direction when following the 5 middle data points and passing by scale 5 in the log-log plots of Figure 6, and (ii) scales 3-4-5 and 5-6-7 provided the FD estimates with the highest *R*^2^-values on average over the 15 miniature conifers, these were chosen for structural complexity analysis and further statistical inference, in which they were respectively denoted FD1 (smaller scales) and FD2 (larger scales); the use of adjusted *R*^2^-values (adjusted for the number of data points) does not change this reasoning. Using the FD1 and FD2 values listed in Table 2 (right columns), we found that the difference between sample means of FD2 and FD1 was very similar in the two groups of coniferous trees: 0.6200 (± 0.0491, *n* = 10) for needlelike-leaf and 0.6290 (± 0.0729, *n* = 5) for scalelike-leaf, and the difference from 0.0 was significantly different at 1% in each group. The last result indicates a greater measured structural complexity for conifer branching patterns at larger scales than at smaller scales.

### Differences in means of shade tolerance index and conifer crown traits

The sample means and associated standard errors of the 12 variables that were studied for the two groups of coniferous trees in relation to their leaf type are presented in Table 3, together with the results of *t*-tests for the statistical comparisons of means. Differences are significant at 5% for STI (greater mean for the needlelike-leaf group), FD1 and FD2 as measures of structural complexity of the branching pattern (greater mean for the scalelike-leaf group), and Ā_D_ prior to standardization by BA, as one of the important crown traits for plant light interception efficiency (greater mean for the needlelike-leaf group). That is, for one third of the variables studied for both groups of conifers, and one or two variables per type of variable. The absence of a significant difference between the two mean values of Ā_D_ after standardization is one of a small number of effects of the standardization that we have observed in our study.

**Table 3:** **Group means depending on leaf type and corresponding standard errors for the shade tolerance index and the crown traits that were measured from CT scanning data and CT images for both groups of miniature conifers, together with the result of the statistical comparison of the two means per variable (AH_0_, Accept the null hypothesis of equality of means; RH_0_, Reject, at 5%)**.

**Variable**	**Needlelike-leaf group mean**	**Standard error (*n* = 10)**	**Scalelike-leaf group mean**	**Standard error (*n* = 5)**	**Probability of significance two-sample *t*-test**
STI	3.55	0.23	2.30	0.37	0.0102 (RH_0_)
LA^†^	12636	1691	11511	1388	0.6737 (AH_0_)
LA^‡^	256	27	318	62	0.2968 (AH_0_)
PCT	0.531	0.035	0.567	0.070	0.6176 (AH_0_)
FD1	1.177	0.030	1.449	0.051	0.0003 (RH_0_)
FD2	1.797	0.069	2.078	0.078	0.0269 (RH_0_)
*V*^†^_L_	125469	19245	82782	25989	0.2167 (AH_0_)
*V*^‡^_L_	2404	242	2044	479	0.4647 (AH_0_)
*V*_L_/*V*_B_	2.088	0.384	2.045	0.698	0.9542 (AH_0_)
Ā^†^_D_	44253	2157	34845	3192	0.0275 (RH_0_)
Ā^‡^_D_	920	67	935	127	0.9086 (AH_0_)
BA	51.7	6.2	40.2	6.3	0.2171 (AH_0_)

For the definition of abbreviations, see Table 1.

† Not standardized,

‡ Standardized by basal area.

### Relations to shade tolerance and among crown traits

Several of the significant correlations observed were expected, such as the positive ones: (i) between FD1 and FD2 (when structural complexity of branching pattern is higher/lower over smaller scales, it is higher/lower over larger scales), (ii) between *V*_L_ and *V*_L_/*V*_B_ (by construction), and (iii) between a size trait and itself, not standardized vs. standardized by BA (assuming or anticipating that the distribution of BA values among the 15 miniature conifers would be almost uniform), and (iv) the negative correlation between STAR and *A*_L_ (by construction). Besides those correlations, it is worth commenting on the following relationships found: (i) correlations between STI and FD1, FD2 are both significant and negative for the needlelike-leaf group, but both significant and positive for the scalelike-leaf group; (ii) correlation between STI and Ā_D_ is not significant for the first group, but is positive and significant at 5% prior to standardization by BA for the second group; and (iii) several correlations between branching complexity measures FD1, FD2, and leaf areas (average displayed or in vertical projection of the crown, absolutely or relatively) are significant and positive for the scalelike-leaf group, whereas it is rather with leaf and branch volumetric measures that FD1 and FD2 are correlated significantly and negatively for the needlelike-leaf group. All the significant correlations, at 5 or 10%, are clearly identified in Tables 4, 5. The correlations of Ā_D_ with STI and FD1, which were both positive and significant at 5% prior to standardization of Ā_D_ by BA, remained positive (0.5270 and 0.4000, respectively) but lost their statistical significance after standardization of Ā_D_ by BA (see Table 5B); the sample size of the scalelike-leaf group (*n* = 5) makes the discussion of this result difficult to pursue, except to say that the BA measures were not uniformly distributed in that group.

**Table 4:** **Spearman's rank-based correlation coefficients (with probabilities of significance below, in parentheses) between the shade tolerance index and crown traits measured from CT scanning data and CT images appropriately processed, by group depending on leaf type (*a*) needlelike (*n* = 10) and (*b*) scalelike (*n* = 5); the correlations statistically significant at 5% are bolded and underlined; those only significant at 10% are simply underlined. For the definition of abbreviations, please see Table 1**.

**Variable**	**LA^†^**	**PCT**	**FD1**	**FD2**	***V*_L_**^†^	***V*_L_**/*V*_B_	***N*^†^**		***Ā***_D_ ^†^	***A*_L_**^†^	**STAR**
**(A)**											
STI	0.3687 (0.2945)	0.0127 (0.9722)	−0.5594 (0.0927)	**−0.8772** (0.0009)	0.0318 (0.9305)	0.3496 (0.3221)	**−0.6420** (0.0454)		0.2987 (0.4018)	−0.0127 (0.9722)	0.3178 (0.3708)
LA^†^		−0.1030 (0.7770)	−0.5030 (0.1383)	−0.4424 (0.2004)	0.2606 (0.4671)	0.2485 (0.4888)	−0.0182 (0.9602)		0.5758 (0.0816)	0.2485 (0.4888)	−0.0545 (0.8810)
PCT			0.0545 (0.8810)	0.1515 (0.6761)	0.6242 (0.0537)	0.0788 (0.8287)	0.6242 (0.0537)		0.6121 (0.0600)	0.5879 (0.0739)	**−0.6485** (0.0425)
FD1				**0.7576** (0.0111)	−0.2848 (0.4250)	**−0.7212** (0.0186)	0.2364 (0.5109)		−0.2485 (0.4888)	−0.2364 (0.5109)	0.1151 (0.7514)
FD2					−0.1515 (0.6761)	**−0.6485** (0.0425)	0.6121 (0.0600)		−0.2727 (0.4458)	−0.1030 (0.7770)	−0.2000 (0.5796)
*V*^†^_L_						0.6000 (0.0667)	0.6121 (0.0600)	**0.7091** (0.0217)	**0.9879** (<0.0001)	**−0.9151** (0.0002)
*V*_L_/*V*_B_							−0.0545 (0.8810)		0.2121 (0.5563)	0.5394 (0.1076)	−0.4061 (0.2443)
*N*^†^									0.4303 (0.2145)	**0.6485** (0.0425)	**−0.8424** (0.0022)
Ā^†^_D_										**0.6970** (0.0251)	−0.5394 (0.1076)
*A*^†^_L_											**−0.9273** (0.0001)
**Variable**	**LA^†^**	**PCT**	**FD1**	**FD2**	***V*_L_**^†^	***V*_L_**/*V*_B_	**Ā_D_**^†^				
**(B)**											
STI	0.7906 (0.1114)	0.2108 (0.7336)	**0.9487** (0.0138)	0.0527 (0.9329)	0.3689 (0.5411)	0.0000 (1.0000)	**0.9487** (0.0138)				
LA^†^		0.1000 (0.8729)	**0.9000** (0.0374)	0.2000 (0.7471)	−0.2000 (0.7471)	−0.5000 (0.3910)	0.7000 (0.1881)				
PCT			0.2000 (0.7471)	**0.9000** (0.0374)	0.5000 (0.3910)	0.3000 (0.6238)	0.5000 (0.3910)				
FD1				0.1000 (0.8729)	0.1000 (0.8729)	−0.3000 (0.6238)	**0.9000** (0.0374)				
FD2					0.2000 (0.7471)	0.1000 (0.8729)	0.3000 (0.6238)				
*V*^†^_L_						**0.9000** (0.0374)	0.5000 (0.3910)				
*V*_L_/*V*_B_							0.1000 (0.8729)				

† Not standardized by basal area.

**Table 5:** **Spearman's rank-based correlation coefficients (with probabilities of significance below, in parentheses) between the shade tolerance index and crown traits measured from CT scanning data and CT images appropriately processed, by group depending on leaf type (*a*) needlelike (*n* = 10) and (*b*) scalelike (*n* = 5); the correlations statistically significant at 5% are bolded and underlined; those only significant at 10% are simply underlined**.

**Variable**	**LA^‡^**		***V*^‡^_L_**	***N*^‡^**	**Ā_D_**^‡^		***A*_L_**^‡^
**A**							
STI	0.3115 (0.3810)		0.0826 (0.8205)	**−0.7310** (0.0163)	0.3623 (0.3035)		−0.1462 (0.6869)
LA^†^	**0.7697** (0.0092)		0.1151 (0.7514)	−0.2000 (0.5796)	−0.1273 (0.7261)		0.1394 (0.7009)
PCT	−0.5394 (0.1076)		0.1758 (0.6272)	0.1515 (0.6761)	−0.5151 (0.1276)		0.2242 (0.5334)
FD1	−0.4788 (0.1615)		−0.3697 (0.2931)	0.1394 (0.7009)	−0.2485 (0.4888)		−0.3091 (0.3848)
FD2	−0.3818 (0.2763)		−0.1394 (0.7009)	**0.6606** (0.0376)	−0.3212 (0.3655)		0.0667 (0.8548)
*V*^†^_L_	−0.0182 (0.9602)		**0.6970** (0.0251)	0.2848 (0.4250)	−0.3818 (0.2763)		**0.6606** (0.0376)
*V*_L_/*V*_B_	0.1758 (0.6272)		0.5030 (0.1383)	−0.1273 (0.7261)	0.0667 (0.8548)		0.3576 (0.3104)
*N*^†^	−0.2727 (0.4458)		0.3454 (0.3282)	**0.7697** (0.0092)	−0.5515 (0.0984)		0.5758 (0.0816)
Ā^†^_D_	0.0667 (0.8548)		0.3091 (0.3848)	−0.0182 (0.9602)	−0.3091 (0.3848)		0.3454 (0.3282)
*A*^†^_L_	0.0182 (0.9602)		**0.7333** (0.0158)	0.3576 (0.3104)	−0.3697 (0.2931)		**0.7212** (0.0186)
STAR	0.1515 (0.6761)		**−0.6727** (0.0330)	−0.6121 (0.0600)	0.4545 (0.1869)		**−0.7454** (0.0133)
LA^‡^			0.2727 (0.4458)	−0.0667 (0.8548)	0.1879 (0.6032)		0.2364 (0.5109)
*V*^‡^_L_				0.5030 (0.1383)	0.2485 (0.4888)		**0.9273** (0.0001)
*N*^‡^					−0.0667 (0.8548)		**0.7333** (0.0158)
Ā^‡^_D_							0.1515 (0.6761)
**Variable**		**LA^‡^**		***V*_L_**^‡^		**Ā_D_**^‡^	
**B**							
STI		0.5797 (0.3056)		0.7379 (0.1546)		0.5270 (0.3615)	
LA^†^		0.7000 (0.1881)		0.2000 (0.7471)		0.2000 (0.7471)	
PCT		−0.6000 (0.2848)		0.1000 (0.8729)		−0.6000 (0.2848)	
FD1		0.6000 (0.2848)		0.5000 (0.3910)		0.4000 (0.5046)	
FD2		−0.5000 (0.3910)		−0.2000 (0.7471)		−0.8000 (0.1041)	
*V*^†^_L_		−0.3000 (0.6238)		0.8000 (0.1041)		0.2000 (0.7471)	
*V*_L_/*V*_B_		−0.4000 (0.5046)		0.6000 (0.2848)		0.1000 (0.8729)	
Ā^†^_D_		0.3000 (0.6238)		0.7000 (0.1881)		0.3000 (0.6238)	
LA^‡^				0.3000 (0.6238)		0.7000 (0.1881)	
*V*^‡^_L_						0.7000 (0.1881)	

For the definition of abbreviations, please see Table 1.

† Not standardized, ‡Standardized by basal area.

‡ Standardized by basal area.

### Light interception efficiency for needlelike-leaf group

The variables total tree leaf number, total tree leaf area and average silhouette-to-total-area ratio were studied for the 10 individuals of the needlelike-leaf group, because individual leaves could be isolated from CT scanning data for them. The corresponding sample means and standard errors are: *N* (not standardized), 3857 ± 724; *N* (standardized), 76 ± 12; *A*_L_ (not standardized), 253,106 ± 38,810; *A*_L_ (standardized), 4884 ± 502; and STAR, 20.53 ± 2.42.

**Figure 7:**
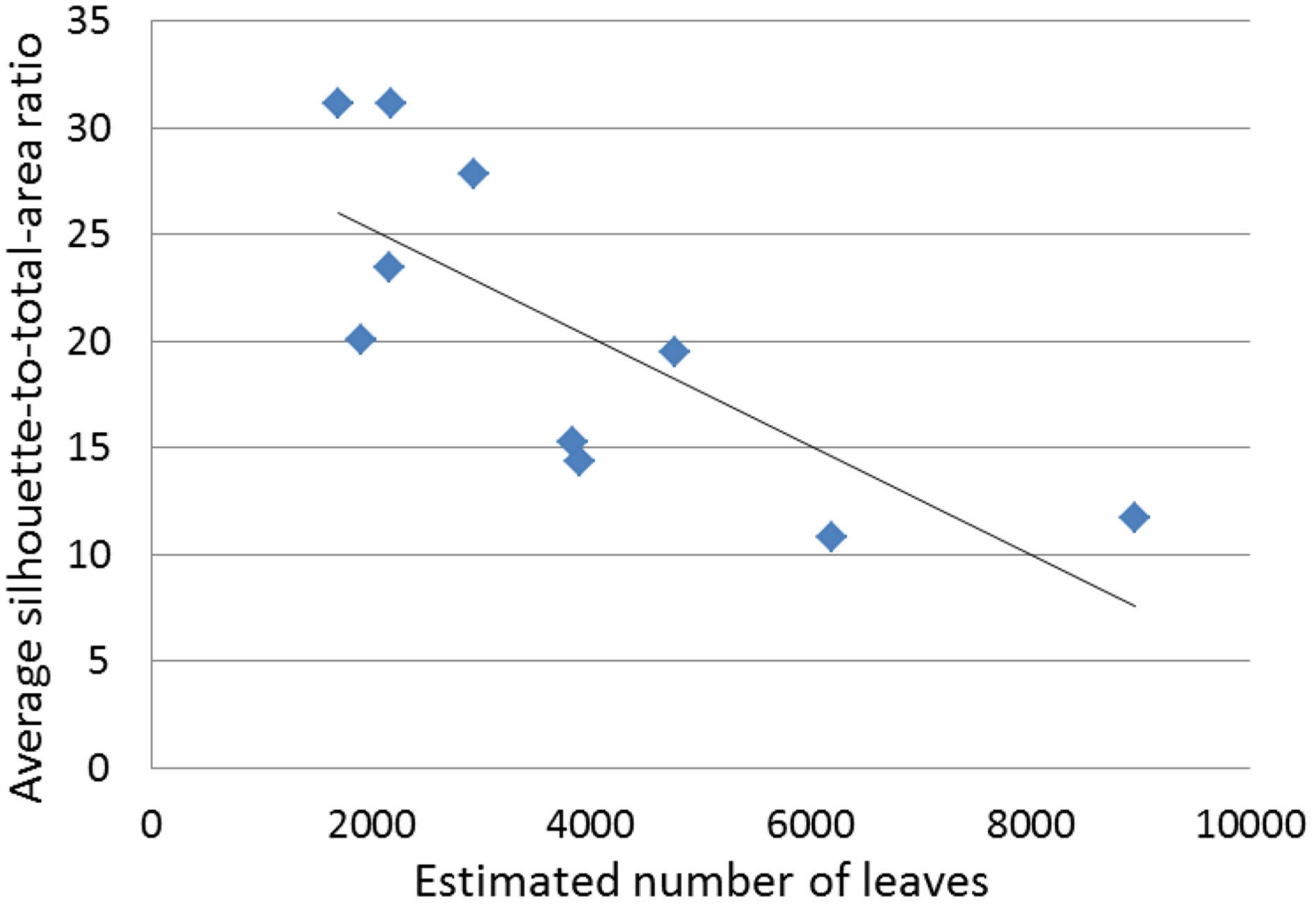
**Average silhouette-to-total-area ratio (**STAR**) against estimated number of leaves (*N*) for the 10 miniature conifers with needlelike leaves**.

Significant correlations of STAR with other variables, at 5 or 10%, are all negative: regardless of standardization, with PCT; before and after standardization by BA, with *V*_L_, *N* and *A*_L_. The scattergram of STAR against *N* (not standardized), using the 10 data points obtained in our study, shows indeed a strong negative relationship (Figure 7); this could be expected (Duursma et al., 2012, Figure 7A), but had not yet been observed with numbers of leaves in the thousands.

## Discussion

### CT scanning technology vs. other approaches to 3-D tree crown reconstruction

The CT scanning of the crown of any of the 15 miniature conifers in our study generated around 125 million CT numbers (Subsection Computed Tomography Scanning). This numerical CT scanning data, which is made of indirect measures of density of all the parts of the crown (stem, branches, and leaves) and the surrounding air, can be mapped in 512 × 512 CT images with lighter and darker gray tones for pixels with higher and lower densities (see examples in Figures 3A,B, left panels). Even more interestingly, 3-D images of complete crowns can be constructed with branches colored in brown and leaves in green (Figure 5); skeletons of branching patterns (Figure 4) are first extracted from the CT images and then let “grow” in an iterative procedure, only to draw the limits between the end of a branch and the beginning of a leaf or an area with leaves, for which CT numbers are available too. After CT scanning, we did not proceed to destructive sampling, which would have consisted in detaching the leaves from the branches as in Foroutan-pour et al. (1999a) who studied soybean canopies without a CT scanner. In our case, there is an intrinsic difficulty of defoliating conifers with sufficient accuracy, especially those with scalelike leaves.

Other approaches, procedures and techniques have been used for 3-D crown reconstruction, but for deciduous trees (e.g., hybrid poplar, sugar maple, yellow birch), generally potted 2–3 year-old saplings, and a cactus-like euphorbia tree. For example, Delagrange and Rochon (2011) worked with a hybrid poplar clone grown in a nursery for 2 years, to compare the results obtained from high-definition photographs in the Tree Analyser (TA) software using a space carving approach vs. 3-D “point clouds” acquired from terrestrial light detection and ranging (T-LiDAR) scans performed on trees without leaves to reconstruct the lignified structure of the sapling, to which foliage was added using allometric relationships between the number of leaves and maximum leaf length and the length of the current-year shoot. Even though some discrepancy is visible between the crown in the black-and-white picture in Figure 1A of Delagrange and Rochon (2011) and the crown reconstructed from T-LiDAR scans in their Figure 2D, the authors found that T-LiDAR is better than TA in terms of precision and accuracy of the reconstruction. The discrepancy is likely to be the result of performing T-LiDAR scanning of the crown after leaves had been detached from it; this could have implications for the results of fractal analysis (structural complexity) and light interception efficient analysis (space occupancy). Using portable scanning LiDAR data, Hosoi et al. (2013) developed a method to produce a 3-D voxel-based solid model of a tree (voxel size: 0.5 × 0.5 × 0.5 cm^3^), for accurate estimation of the volume of woody material. They applied their model to a Japanese zelkova tree, and found very satisfactory results for the stem and large branches (diameter > 1 cm); the error in volume estimates was 0.5%.

Investigating an approach for 3-D data collection on plant architecture that would not be time-consuming and would not require costly experiment, Nock et al. (2013) tested a low-cost, 3-D camera and open-source software for the measurement of stem and branch diameters and lengths. Besides technical and calibration aspects, the authors report that the tested camera is able to accurately capture the diameter of maple branches > 6 mm, and a cactus-like euphorbia is well-acquired thanks to the width of its axes. Focusing on tree crown reconstruction from point clouds acquired with terrestrial LiDAR scanning, the study of Delagrange et al. (2014) can be seen as a follow-up to Delagrange and Rochon (2011), with the presentation of an improved skeletal extraction method for use with 104 or 105 points in the cloud for a synthetic tree and real branches of elm and from 3-year-old sugar maple and yellow birch saplings grown under low and high light regimes; some difficulty to detect smaller branches (length < 3.5 cm) could potentially affect the results of a fractal analysis of the branching pattern, whereas the authors declare that small branches account for little in terms of total skeleton length; no results for leaves were reported.

### Space occupancy in crowns of coniferous trees

The skeletal branching patterns constructed from CT scanning data in this study revealed “gaps” in the crowns of coniferous trees that are partially filled to various degrees, depending on species, by needlelike or scalelike leaves (Figures 4, 5). The size of gaps in skeletal branching patterns is related to the level of structural complexity: the more complex the branching patterns (the higher the degree of subdivision of branches), the smaller the gaps, and vice versa; see “Foliage Dispersion” in Valladares and Niinemets (2007, p. 119). We observed this relationship for needlelike-leaf coniferous trees, but not directly on total tree leaf volume. We observed this relationship for that group through a significant and negative correlation between FD1, FD2 and leaf volume-to-branch volume ratio. We did not observe a similar relationship for the scalelike-leaf group, which may be explained by the different type of leaves (i.e., short and pasted on branches vs. longer needles forming a certain angle with the branch) and a different range of FD1, FD2 values (i.e., lower for needlelike-leaf vs. higher for scalelike-leaf; Tables 2, 3). The higher (lower) the value of the fractal dimension parameter, the more (less) complex the structure of the branching pattern.

### Range of shade tolerance index values and the leaf-type classification factor

Our primary goal in conducting this study was not to cover the whole range (1–5) of possible values for the shade tolerance index in each of two groups of miniature conifers classified according to their leaf type. Instead, it was of biophysical nature—the 15 experimental trees were chosen based on the architecture of their crown, among the species and varieties available at the grower at the time. The main research objective was to address technological challenges, including the collection and advanced appropriate processing of CT scanning data for tree crown reconstruction at an unprecedented level, while providing insight on the physiological side—through shade tolerance—and thus preparing for future larger studies on the subject after establishment of the technological and analytical protocol and procedures. Accordingly, it is important to keep in mind the ranges of shade tolerance index values in the present study, when commenting the differences observed between groups in mean values of other crown traits and in the correlations between some of the crown traits in the last subsection of the Discussion. The inclusion of miniature varieties of *Pinus* in the needlelike-leaf group would definitely decrease the mean value of the shade tolerance index value of that group; a presently open question that can be answered in a later study concerns the resulting effects on mean values of other crown traits and related correlations for that group.

### Leaf type effects and observed differences in shade tolerance, crown structure and light interception

Based on the relative 2-D and 3-D measures that are the proportion of leaf area in the vertical projection of the crown and the leaf volume-to-branch volume ratio, the two groups of miniature conifers showed very little difference in mean values, while the corresponding absolute measures showed a greater difference which was not close to be significant (Table 3). Interestingly, the four variables that show significant differences in mean values between groups also show differences in correlations (Tables 3, 4).

In contrast with the rigidity and proximity of scalelike leaves to branches, needles have a well-identified point of attachment to branches in the form of an alveolus and possess a greater mobility around their point of attachment; this cannot explain *per se* the higher mean value of the index for the needlelike-leaf group (see the point raised in Subsection Range of Shade Tolerance Index Values and the Leaf-Type Classification Factor), but may provide a leaf canopy more instrumental in capturing the irradiance in low light environments. The size of leaves seems larger for needles, but we have only visual observations from CT images to use for scalelike leaves.

The differences in mean values of FD1, FD2 indicate that: (i) branches tend to be aligned at smaller scales in 3D (i.e., the mean FD1 is close to 1.0, the dimension of a straight line in classical Euclidean geometry) in the needlelike-leaf group, but are laid in a manner mid-way between linear and planar (i.e., the mean FD1 is close to 1.5, between 1.0 and 2.0, the dimension of a plane) in the scalelike-leaf group; and (ii) over larger scales, the spatial distribution of branches is almost planar (i.e., the mean FD2 is close, but not equal to 2.0) for our miniature conifers with needlelike leaves, and slightly more complex than a planar distribution (i.e., the mean FD2 is greater than 2.0) for those with scalelike leaves. These results concern the crown structures, and reflect a greater flexibility and degree of repeated subdivision of the branching patterns of scalelike-leaf trees, likely to adjust for the characteristics of their leaves and succeed in capturing a sufficient amount of light for their survival and development. The difference in mean values of the average leaf area displayed is consistent with the other differences observed in mean values between groups, and could be expected in some way; the mean value of Ā_D_ is greater for the needlelike-leaf group, that is, the reverse than for FD1 and FD2.

Combining our results on the differences in mean values of the shade tolerance index and FD1 with those on the change in sign of their correlations depending on leaf type (i.e., negative for needlelike and positive for scalelike), a relationship between the two variables that is quadratic and asymmetric, instead of linear or at the least monotonic, seems possible. In fact, while the shade tolerance index takes its highest value of 4.5 for FD1 < 1.1 (two times) in the needlelike-leaf group (minimum FD1: 1.0639, maximum FD1: 1.3465), it takes its highest value of 3.5 for FD1 = 1.5781 after a minimum of 1.5 (two times) for FD1 = 1.2741 and 1.4252 in the scalelike-leaf group (Table 2). A larger sample size for the scalelike-leaf group, including a broader range of shade tolerance index values in that group (Subsection Range of Shade Tolerance Index Values and the Leaf-Type Classification Factor), would allow the assessment of such a quadratic relationship more finely.

## Concluding remarks

So far, a high-resolution CT scanner (non-micro, due to the size of the field of view) has been used in a much smaller number of studies on plant canopy architecture, compared to those conducted on plant root systems using micro or non-micro CT scanning. One of our conclusions is that there could or should be more studies like ours (i.e., for crowns of small-size trees), because the resolution achieved with a X-ray CT scanner of medical type such as a Toshiba XVision is very fine and sufficient at the whole-plant scale (i.e., down to 0.23 × 0.23 × 0.20 mm^3^ and not exceeding 0.62 × 0.62 × 0.4 mm^3^ in our study). Accordingly, detailed graphical and quantitative information could be gathered for two groups of miniature conifers with different leaf types (i.e., needlelike vs. scalelike), regarding their leaf areas and volumes and the complexity of their branching patterns.

Differences between groups in mean values of crown traits measured from CT scanning data and a shade tolerance index obtained separately were assessed statistically. Significant differences were found for shade tolerance, fractal dimensions and the average leaf area displayed. These differences between mean values had implications for correlations; in particular, shade tolerance was negatively correlated with fractal dimensions for the needlelike-leaf group, and positively correlated with one fractal dimension in the miniature coniferous with scalelike leaves studied. These findings were complemented with the acceptance of the hypothesis of bifractality of the branching pattern over the two groups of miniature conifers and the presentation of new documentation for conifers with needlelike leaves about the strong negative relationship between the average silhouette-to-total-area ratio and the number of leaves, when the latter are in large to very large numbers. In closing, our results here, obtained for crowns of miniature conifers analyzed thoroughly and accurately thanks to CT scanning technology and advanced data processing, could be used for crown modeling of non-miniature indigenous species in situations where the leaf size-to-branch length ratio would justify it, for example for juvenile indigenous trees of a size that fits in the gantry of the CT scanner.

### Conflict of interest statement

The authors declare that the research was conducted in the absence of any commercial or financial relationships that could be construed as a potential conflict of interest.
